# Intestinal epithelium-specific Fut2 deficiency promotes colorectal cancer through down-regulating fucosylation of MCAM

**DOI:** 10.1186/s12967-023-03906-0

**Published:** 2023-02-04

**Authors:** Weijun Wang, Xuelian Tang, Caihan Duan, Shuxin Tian, Chaoqun Han, Wei Qian, Xin Jiang, Xiaohua Hou, Rong Lin

**Affiliations:** 1 Department of Gastroenterology, Union Hospital, Tongji Medical College, Huazhong University of Science and Technology, Wuhan, 430022 China; 2 Department of Gastroenterology, The First Affiliated Hospital of Medical College, Shihezi University, Shihezi, 832008 China

**Keywords:** Fut2, MCAM, Fucosylation, Colorectal cancer, CRC

## Abstract

**Background:**

Our previous study showed that fucosyltransferase 2 (Fut2) deficiency is closely related to colitis. Colitis increases the risk for the development of colorectal cancer (CRC). This study aimed to investigate the effect and underlying mechanism of action of Fut2 in CRC.

**Methods:**

Intestinal epithelium-specific Fut2 knockout (*Fut2*^△IEC^) mice were used in this study. CRC was induced using azoxymethane (AOM) and dextran sulfate sodium (DSS). Immunofluorescence was used to examine the fucosylation levels. Proteomics and N-glycoproteomics analyses, Ulex Europaeus Agglutinin I (UEA-I) affinity chromatography, immunoprecipitation, and rescue assay were used to investigate the mechanism of Fut2 in CRC.

**Results:**

The expression of Fut2 and α-1,2-fucosylation was lower in colorectal tumor tissues than in the adjacent normal tissues of AOM/DSS-induced CRC mice. More colorectal tumors were detected in *Fut2*^△IEC^ mice than in control mice, and significant downregulation of melanoma cell adhesion molecule (MCAM) fucosylation was detected in the colorectal tumor tissues of *Fut2*^△IEC^ mice. Overexpression of Fut2 inhibited cell proliferation, invasion and tumor metastasis in vivo and in vitro in SW480 and HCT116 cells. Moreover, fucosylation of MCAM may be a mediator of Fut2 in CRC. Peracetylated 2-F-Fuc, a fucosyltransferase inhibitor, repressed fucosylation modification of MCAM and reversed the inhibitory effects of Fut2 overexpression on SW480 cell proliferation, migration, and invasion. Our results indicate that Fut2 deficiency in the intestinal epithelium promotes CRC by downregulating the fucosylation of MCAM.

**Conclusions:**

The regulation of fucosylation may be an potential therapy for CRC, especially in patients with Fut2 gene defects.

**Supplementary Information:**

The online version contains supplementary material available at 10.1186/s12967-023-03906-0.

## Background

Colorectal cancer (CRC) is the third most commonly diagnosed malignancy and the fourth leading cause of cancer-related deaths worldwide [1–3]. The global morbidity rate is 10.0%, and the mortality rate is 9.4% [4]. CRC includes sporadic, hereditary, and colitis-associated CRC [5]. In addition to somatic mutations and epigenetic changes, accumulating evidence indicates that chronic inflammation is associated with an increased risk of CRC development [6, 7]. Additionally, increasing evidence indicates a strong association between inflammatory bowel disease and CRC [8, 9].

Fucosyltransferase 2 (Fut2) is one of the enzymes responsible for the addition of fucose to proteins or lipids by α-1,2-fucosylation in the intestinal mucosa [10, 11]. Our previous study reported that intestinal epithelium‐specific Fut2 deficiency exacerbates dextran sulfate sodium (DSS)‐induced colitis [12]. However, the relationship between Fut2 expression and CRC remains unclear. Since chronic inflammation is considered a risk factor for CRC development, the role of Fut2 in CRC remains to be further explored.

Fut2, as a vital α-1,2-fucosyltransferase, mainly regulates the fucosylation of cells or specific proteins [13]. For example, our previous study found that Fut2 mediates fucosylation of intestinal epithelial cells to protect intestinal barrier function and relieve intestinal inflammation [12]. In addition, our previous study concluded that FUT2-mediated fucosylation of intestinal epithelial cells protects intestinal barrier function and relieves intestinal inflammation [14]. Other studies have also reported that Fut2 induces lung epithelial fucosylation, thereby exacerbating airway inflammation in asthmatic patients [15]. Regarding specific proteins, one study revealed that a loss of Fut2-mediated mucin O-glycosylation pattern of Muc5ac worsens the gastric mucosal binding of *Helicobacter pylori* BabA adhesin, which is a recognized feature of pathogenicity [16]. In addition, Fut2 can also modulate the glycosylation profile of mucus secreted by HT-29 cells, thereby regulating the intestinal epithelial barrier functions [17]. Thus, our objective was to determine whether Fut2 is involved in CRC by regulating the fucosylation of certain key proteins.

In this study, intestinal epithelium-specific Fut2 knockout mice (*Fut2*^△IEC^ mice) were generated to investigate the role of Fut2 in CRC. N-glycoproteomics and proteomics analyses were conducted to identify specific proteins regulated by Fut2. Ulex Europaeus Agglutinin I (UEA-I) affinity chromatography, immunoprecipitation, and rescue assays were performed to investigate the underlying mechanism of action of Fut2 in CRC. Collectively, our results revealed that intestinal epithelium-specific Fut2 deficiency mice were susceptible to azoxymethane/dextran sulfate sodium (AOM/DSS)-induced CRC by inhibiting the fucosylation of melanoma cell adhesion molecule (MCAM).

## Methods

### Mice

As our previous study reported, we used Pvillin-Cre recombinase transgenic C57BL/6 mice (Pvillin-Cre TG mice) and *Fut2*^flox/flox^ C57BL/6 mice (purchased from GemPharmatech Co. Ltd) to cross and generate mice with the *Fut2* gene specifically deleted in intestinal epithelial cell (Pvillin-Cre + *Fut2*^flox/flox^ mice, abbreviated as *Fut2*^△IEC^) [12]. Knock-out of *Fut2* in intestinal epithelial cells and the loss of α1,2-fucosylation in the tissue were validated in our previous study [12]. *Fut2*^△IEC^ male mice (8–10 weeks old) were used in further experiments. Fut2 negative mice (wild-type (WT) mice) were used as controls for *Fut2*^△IEC^ mice. All mice were housed in the specific pathogen-free (SPF) grade facility of Huazhong University of Science and Technology and maintained under 12 h light/dark cycles with free access to food and water [12]. All animal studies were approved by the Animal Experimentation Ethics Committee of Huazhong University of Science and Technology and performed in accordance with national and EU guidelines [12].

### Model establishment

WT and *Fut2*^△IEC^ mice were randomly divided into four groups: WT control, WT AOM/DSS, *Fut2*^△IEC^ control, and *Fut2*^△IEC^ AOM/DSS (n = 5 per group). Mice in the AOM/DSS groups (WT AOM/DSS and *Fut2*^△IEC^ AOM/DSS) and AOM/DSS-induced colitis-associated CRC models were prepared as previously described [18]. Briefly, the male mice were first treated with AOM (10 mg/kg). One week later, the mice were fed 2% DSS for 1 week, followed by regular drinking water for 2 weeks. This cycle was repeated three times. Throughout the trial, body weight, diarrhea, and macroscopic bleeding of the mice were monitored.

### Histological examination

For histological examination, distal colon specimens were fixed in 4% formalin for 24 h and embedded in paraffin, stained with hematoxylin and eosin (H&E), and analyzed by a pathologist that had no prior knowledge of the experimental procedures.

### Cell culture and treatment

Two CRC cell line (SW480 cells and HCT116 cells) were used in this study. SW480 cells were cultured in Roswell Park Memorial Institute (RPMI) 1640 medium supplemented with 10% fetal bovine serum (FBS) and 1% penicillin-streptomycin [19]. HCT116 cells were grown in Dulbecco’s Modified Eagle Medium (DMEM) supplemented with 10% FBS and 1% penicillin-streptomycin [19]. Cells were maintained in a 5% CO_2_ incubator at 37 °C. The cells were routinely tested to exclude mycoplasma contamination.

### Lentivirus transfection

The lentiviral vector system and the empty vectors were purchased from the GeneChem Corporation (Shanghai, China). Cells (5 × 10^5^) were transfected with the specific virus at a multiplicity of infection of 20 in the presence of polybrene (5 μg/mL). After 12 h, the supernatant was replaced with the cultured medium. Expression of Fut2 and MCAM in transfected cells was validated by western blot.

### CCK8 assay

Cell proliferation was assessed using the CCK8 assay kit (Dojindo Laboratories, Kumamoto, Japan). The cells were seeded into 96-well plates at a density of 2 × 10^3^ well^−1^ and incubated in a 5% CO_2_ incubator for 24, 48, or 72 h. Next, 10 μL of CCK8 reagent was added to each well and the plates were returned to the incubator for another 2 h. The absorbances was measured at 450 nm using a microplate reader (BioTek, Winooski, VT, USA).

### Wound-healing assay

Cells were seeded in 6-well plates at 37 °C in a 5% CO_2_ atmosphere until the cells grew to 80% of the coverage area at the bottom of the wells. Straight lines were drawn on the cell layer with a 10 μL pipette tip. The treated 6-well plates were observed and photographed under a microscope at different times.

### Migration and invasion assays

Cell migration and invasion assays were performed in a Transwell chamber (Corning, Corning, NY, USA) according to the manufacturer’s protocol. For the migration assay, 1 × 10^5^ cells were seeded into the upper chamber containing FBS-free medium, and the lower chamber contained complete cell culture media. After incubation at 37 °C in an atmosphere of 5% CO_2_ for the indicated hours, the non-migrating or non-invading cells in the upper chamber were removed with cotton swabs. The cells that penetrated the membrane filters were fixed in 4% methanol, stained with crystal violet, and counted under a light microscope. For the invasion assays, the upper chambers were precoated with 15 μg/μL Matrigel (Corning), following the same procedures as the migration assay.

### Subcutaneous tumorigenesis assay

Twelve BALB/c nude mice (male, 4 weeks-old, weighting 18 ± 2 g) were randomly divided into two groups of six animals each. SW480 and HCT116 cells were suspended in phosphate buffered saline (PBS) at a density of 1 × 10^7^/ml. Next, 200 μL of cell suspension was subcutaneously injected into the axillae to induce tumor growth. After 4 weeks, the mice were sacrificed and their tumor sizes and weights were measured.

### Co-immunoprecipitation (CoIP) assay

Cells were harvested and lysed in 500 μL of CoIP buffer containing a protease inhibitor cocktail (Thermo Fisher Scientific). After centrifugation, cell lysates were collected and precleared by incubating with 20 μL of immobilized UEA-I beads for 1 h at 4 °C. The beads were then discarded using a magnetic frame and the lysates were incubated with a primary antibody or control immunoglobulin G on a rotator at 4 °C overnight. On the following day, 20 μL of immobilized protein A/G beads was added to precipitate the protein complex at 4 °C for 4 h. Subsequently, the samples were washed five times, the beads were boiled in loading buffer, and the proteins were prepared for immunoblot analysis.

### Immunofluorescence and immunohistochemistry

For immunofluorescence staining, paraffin embedded sections (5 μm) with colon tissues were hydrated, treated for antigen retrieval with citrate buffer (pH 6), and the slides of cells were fixed in 4% formalin for 30 min and then washed with PBS for 3 times. For Ulex europaeus agglutinin-I (UEA-I) staining, sections were incubated with rhodamine UEA-I for 1 h at 37 °C. The nuclei were stained with DAPI (Beyotime Biotech, China) for 8 min at room temperature. Images were acquired using a confocal microscope (Nikon, Japan).

Immunohistochemistry (IHC) of colon tissues was performed using a VECTASTAIN Elite ABC kit and DAB Detection kit (Boster Biological Technology Co., Ltd) following the manufacturer’s instructions, using an anti-F4/80 antibody (ARG55738, Arigo).

### Western blot analysis

Proteins were harvested from cells and colon tissues using RIPA Lysis Buffer (Beyotime, Hainan, Jiangsu, China) supplemented with phenylmethyl sulfonyl fluoride protease and phosphatase inhibitors. The total protein concentration was determined using a Pierce^™^ BCA Protein Assay Kit (Thermo Fisher, Waltham, Massachusetts, USA), and denatured protein samples of appropriate quality were subjected to sodium dodecyl sulfate polyacrylamide gel electrophoresis and then transferred to polyvinylidene fluoride membranes. Then membranes were later blocked with 5% skimmed milk and incubated with specific antibodies against Fut2 (Santa Cruze, CA, USA), MCAM (Santa Cruze, CA, USA), actin (Antgene, Wuhan, China), and GAPDH (Antgene, Wuhan, China) overnight at 4 °C. The secondary antibody was purchased from GeneTex (Irvine, California, USA). Protein bands were visualized by the FluorChem Imaging System (ProteinSimple, San Jose, California, USA) using the commercial Pierce^™^ Fast Western Blot Kit and ECL Substrate (Thermo Fisher, Waltham, Massachusetts, USA).

### RNA extraction and qPCR

RNA was extracted from colon tissue or cells using TRIzol reagent (Invitrogen) according to the manufacturer’s protocol. Reverse transcription (cDNA) was synthesized from 1 μg of total RNA with Prime Script RT Master Mix (Takara Biotechnology, Dalian, China). Quantitative Real-time PCR (qPCR) was performed using 1 μl of first-strand cDNA with the LightCycler^®^ 480 SYBR I Master Mix (Roche, Switzerland) at a final volume of 10 μl. All samples were run in triplicate and underwent 45 amplification cycles on a Roche LightCycler R480 (Roche, Switzerland). The relative fold-change in mRNA expression was measured by using the 2^−ΔCT^ method and normalized. The primers used are listed in Additional file 1: Table S1.

### TCGA database

We collected mRNA expression profiles and clinical data of patients with CRC from the Cancer Genome Atlas (TCGA) database (https://tcga-data.nci.nih.gov/tcga/). Clinical data such as gender, age, race, T, N, M stage, pathologic stage, and primary therapy outcome, were also downloaded from TCGA data portal.

### Statistical analysis

The SPSS 20.0, Graphpad prism software and Image J software were used for statistical analysis. Data were presented as mean values ± SEM for independent experiments. For comparison between two groups, a paired t-test was performed. Multiple group comparisons were calculated by one-way analysis of variance (ANOVA). P < 0.05 was considered statistically significant. Proteomic analysis are listed in Additional file 2.

## Results

### Fut2 is downregulated in CRC and correlates with poor prognosis

**Fig. 1:**
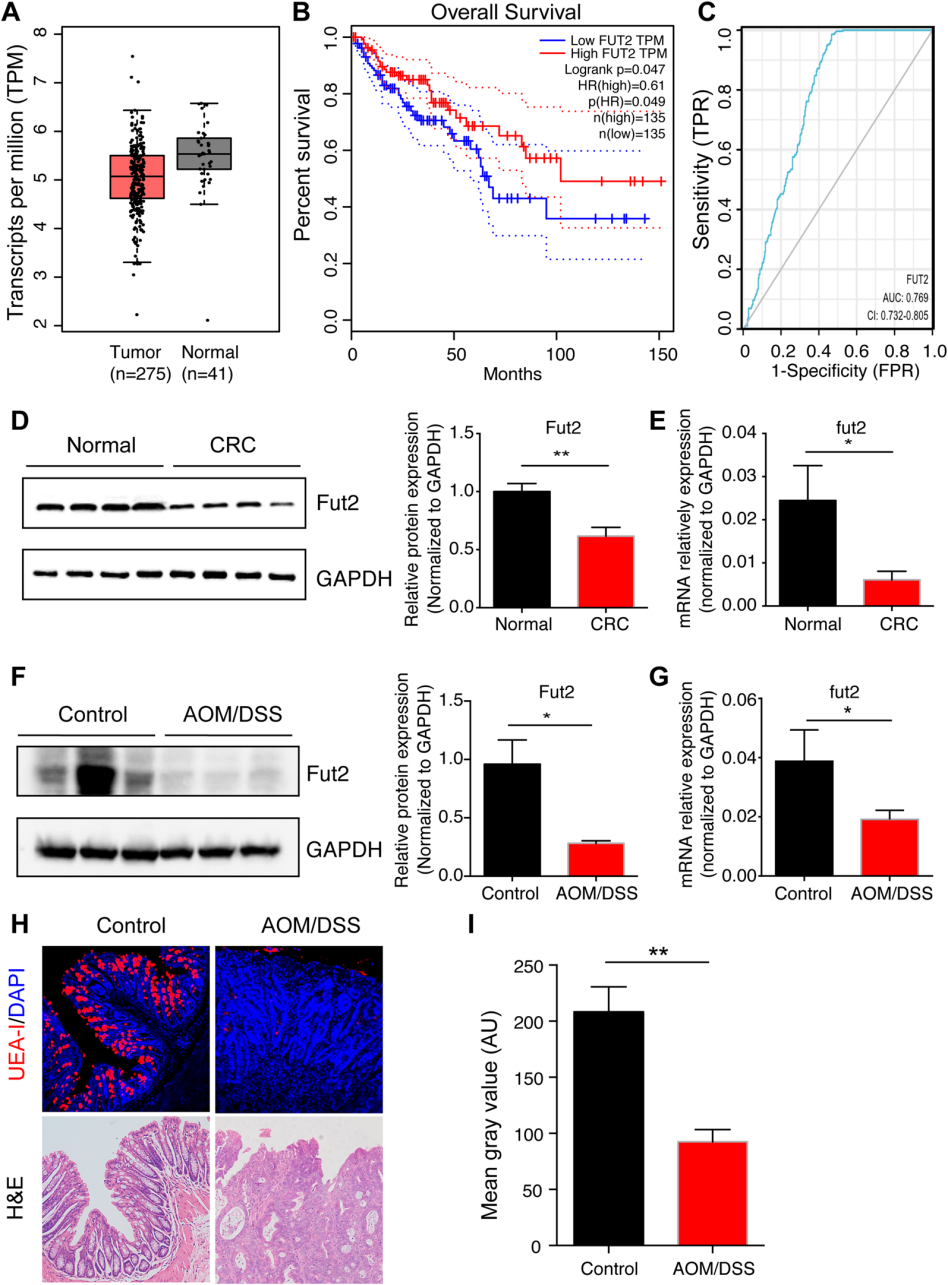
Fut2 is down regulated in CRC and correlates with poor prognosis. **A** Expression of Fut2 mRNA (including 275 CRC tissues and 45 normal colon tissues) from TCGA database was first analyzed. **B** Amplification of Fut2 gene copy number associated with good disease-free survival in TCGA cohort (n = 135, log-rank test, P = 0.047). **C** ROC curves for classifying CRC tissues versus normal colon tissues in the TCGA database. **D** The proteins level of Fut2 in colon tissues extracted from normal and colorectal cancer patient (n = 4 per group). **E** The relative mRNA expression of Fut2 in control and colorectal cancer patient colon tissues was detected by qPCR (n = 4 for per group). **F** The proteins level of Fut2 in colon tissues extracted from control mice and AOM/DSS mice (n = 3 per group). **G** The relative mRNA expression of Fut2 in control and AOM/DSS mice colon tissues was detected by qPCR (n = 5 for per group). **H** The typical images of mice colon tissues stained with UEA-I. **I** The mean gray value of UEA-I in control mice and AOM/DSS mice (n = 5 per group). Data are expressed as mean ± SEM. The data come from three independent experiments. In all panels: *p < 0.05, **p < 0.01. (*CRC* colorectal cancer, *TCGA* The Cancer Genome Atlas, *AOM/DSS* azoxymethane/dextran sodium sulfate, *UEA-I* Ulex Europaeus Agglutinin-I, *SEM* Standard Error of Mean)

Data (275 CRC tissues and 45 normal colon tissues) from TCGA database were analyzed and revealed that Fut2 expression was reduced in CRC tissues compared to non-tumor tissues (Fig. 1A). Furthermore, low expression of Fut2 was correlated with poor prognosis in CRC patients (Fig. 1B). As shown in Table 1, low expression of Fut2 was significantly associated with pathologic stage (stage I-II vs stage III-IV, p = 0.045) and M stage (p = 0.021). Furthermore, the ROC curve indicated that Fut2 expression had good predictive power, with an area under the curve (AUC) of 0.769 (95% confidence interval [CI] = 0.732–0.805), to discriminate CRC tissues from normal tissues (Fig. 1C). Meanwhile, mRNA and protein levels of Fut2 in tumor tissues and adjacent tissues from CRC patients (Fig. 1D, E) and AOM/DSS-induced CRC mice (Fig. 1F, G) were detected using qPCR and western blotting. The results showed that Fut2 mRNA and protein levels were significantly downregulated in tumor tissues compared to the adjacent tissues in both humans and mice (p < 0.05). Moreover, α-1,2-fucosylation in colonic tissues was evaluated using UEA-I staining. As it is shown in Fig. 1H, I, α-1,2-fucosylation of colonic tissues decreased prominently in AOM/DSS mice compared to that in the control mice (p = 0.0014).

**Table 1:** Clinicopathological characteristics of high- and low-Fut2 expression groups

Characteristic	Low expression of Fut2	High expression of Fut2	p
n	239	239	
Age, n (%)			0.113
< = 65	88 (18.4%)	106 (22.2%)	
> 65	151 (31.6%)	133 (27.8%)	
Gender, n (%)			1.000
Female	113 (23.6%)	113 (23.6%)	
Male	126 (26.4%)	126 (26.4%)	
Race, n (%)			**0.026**
Asian	2 (0.7%)	9 (2.9%)	
Black or African American	39 (12.7%)	24 (7.8%)	
White	124 (40.5%)	108 (35.3%)	
T stage, n (%)			0.426
T1	4 (0.8%)	7 (1.5%)	
T2	39 (8.2%)	44 (9.2%)	
T3	161 (33.8%)	162 (34%)	
T4	35 (7.3%)	25 (5.2%)	
N stage, n (%)			0.168
N0	133 (27.8%)	151 (31.6%)	
N1	56 (11.7%)	52 (10.9%)	
N2	50 (10.5%)	36 (7.5%)	
M stage, n (%)			**0.021**
M0	165 (39.8%)	184 (44.3%)	
M1	42 (10.1%)	24 (5.8%)	
Pathologic stage, n (%)			0.078
Stage I	38 (8.1%)	43 (9.2%)	
Stage II	85 (18.2%)	102 (21.8%)	
Stage III	68 (14.6%)	65 (13.9%)	
Stage IV	42 (9%)	24 (5.1%)	
Primary therapy outcome, n (%)			0.186
PD	12 (4.8%)	13 (5.2%)	
SD	2 (0.8%)	2 (0.8%)	
PR	10 (4%)	3 (1.2%)	
CR	96 (38.4%)	112 (44.8%)	
Age, median (IQR)	70 (58, 77)	68 (59, 78)	0.696

Bold values denote two-sided p < 0.05

*Fut2* Fucosyltransferase 2, *PD* partial response, *SD* stable disease, *PR* partial response, *CR* complete response

### Fut2 deficiency enhances promotion of AOM/DSS-induced CRC

**Fig. 2:**
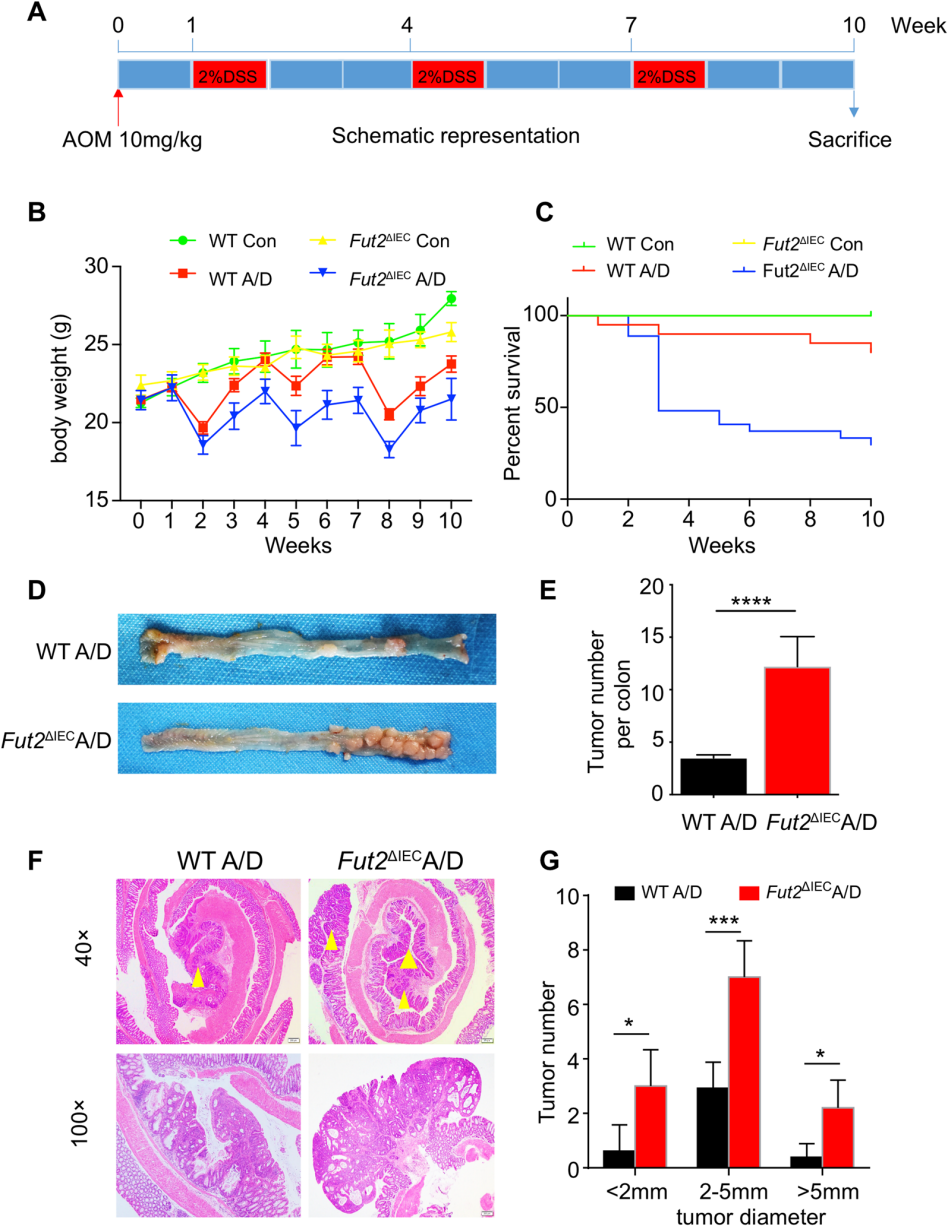
Fut2 deficiency enhances promotion of AOM/DSS-induced CRC. **A** Schematic overview of this colon carcinogenesis model. **B** Body weight change during the disease process. **C** Survival rate of mice from each group. **D** Macroscopical changes in colon. Colons were removed at day 70 from treated WT and *Fut2*^△IEC^ mice, and representative results from 5 independent animals are shown here. **E** Tumor numbers of each group. **F** The typical images of mice colon tissues from treated WT and *Fut2*^△IEC^ mice. **G** The number of tumors corresponding to different tumor sizes of each group. Data are expressed as mean ± SEM. The data come from three independent experiments. In all panels: ***p < 0.001, ****p < 0.0001. (*CRC* colorectal cancer, *AOM/DSS* azoxymethane/dextran sodium sulfate, *WT* wild type, *SEM* Standard Error of Mean)

*Fut2*^△IEC^ mice were used to investigate the role of Fut2 in CRC in *vivo*. *Fut2*^△IEC^ and control mice were intraperitoneally injected with AOM and subjected to three cycles of DSS (Fig. 2A). *Fut2*^△IEC^ mice were susceptible to AOM/DSS compared with WT mice, which mainly manifested as significant weight loss (p < 0.001; Fig. 2B) and high mortality (p = 0.0029) (Fig. 2C). *Fut2*^△IEC^ mice showed a dramatic increase in tumor multiplicity compared with control mice (Fig. 2D); the mean tumor number was 12.2 in *Fut2*^△IEC^ mice versus 3.5 in controls (p < 0.001; Fig. 2E, F). The increase in tumor burden in *Fut2*^△IEC^ mice was mediated by an increase in both the number of small (< 2 mm), medium (2–5 mm), and large (˃ 5 mm) tumors (Fig. 2G).

### Fut2 inhibits cell proliferation, migration and invasion of SW480 and HCT116 cells

**Fig. 3:**
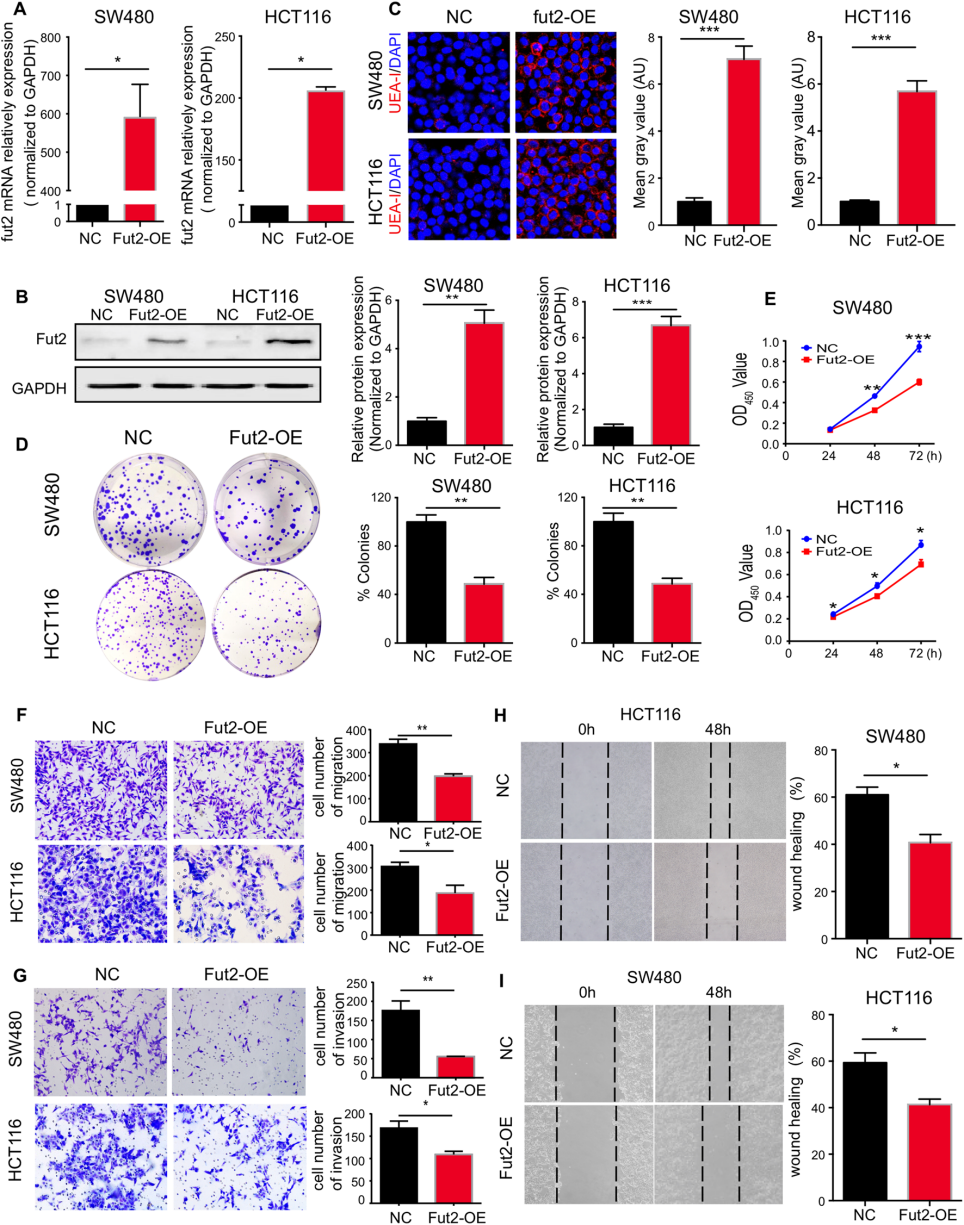
Fut2 inhibits cell proliferation of SW480 and HCT116 cells. **A** The relative mRNA expression of Fut2 in control and fut2 overexpressed SW480 and HCT116 cells was detected by qPCR (n = 3 for per group). **B** The relative protein expression of Fut2 in control and fut2 overexpressed SW480 and HCT116 cells was detected by western blot (n = 3 for per group). **C** The typical images of SW480 and HCT116 cells stained with UEA-I. **D** The plate clone formation experiment of SW480 and HCT116 cells in control and Fut2-overexpression group. **E** CCK8 assays of SW480 and HCT116 cells in control and Fut2-overexpression group. **F** The typical images of transwell migration assay in control and fut2 overexpressed SW480 and HCT116 cells. **G** The typical images of transwell matrigel invasion assay in control and fut2 overexpressed SW480 and HCT116 cells. **H**, **I** Wound-healing assay at 48 h in control and fut2 overexpressed SW480 and HCT116 cells. Data are expressed as mean ± SEM. The data come from three independent experiments. In all panels: *p < 0.05, ***p < 0.001. (*CCK8* Cell Counting Kit-8, *SEM* Standard Error of Mean)

*Fut2* was overexpressed in SW480 and HCT116 cells in vitro to investigate its role in proliferation, migration and invasion of colon cancer cells. We ectopically overexpressed *Fut2* in SW480 and HCT116 cell lines, and the efficiency of overexpression was verified by qPCR and western blotting (Fig. 3A, B). As expected, overexpression of Fut2 also resulted in increased α-1,2-fucosylation in the cells (Fig. 3C).

The mean colony number significantly decreased in SW480 and HCT116 cells overexpressing Fut2 compared to that in control cells (Fig. 3D). CCK8 assays also showed that Fut2 overexpression significantly inhibited SW480 and HCT116 cell proliferation (Fig. 3E). To examine whether migration could be regulated by *Fut2* in CRC cell lines, wound healing and Transwell migration assays were performed. Fut2 overexpression markedly inhibited the migration of SW480 and HCT116 cells. Transwell migration assays revealed that Fut2 overexpression decreased SW480 cell migration by 41% and decreased HCT116 cell migration by 37% (Fig. 3F). In addition, our results showed that invading Fut2-overexpression in SW480 and HCT116 cells decreased significantly compared to that of the control cells (p = 0.0088 and p = 0.027, respectively) (Fig. 3G). This result was also confirmed by a wound-healing assay (Fig. 3H, I).

### Fut2 inhibits tumor proliferation in xenograft model

**Fig. 4:**
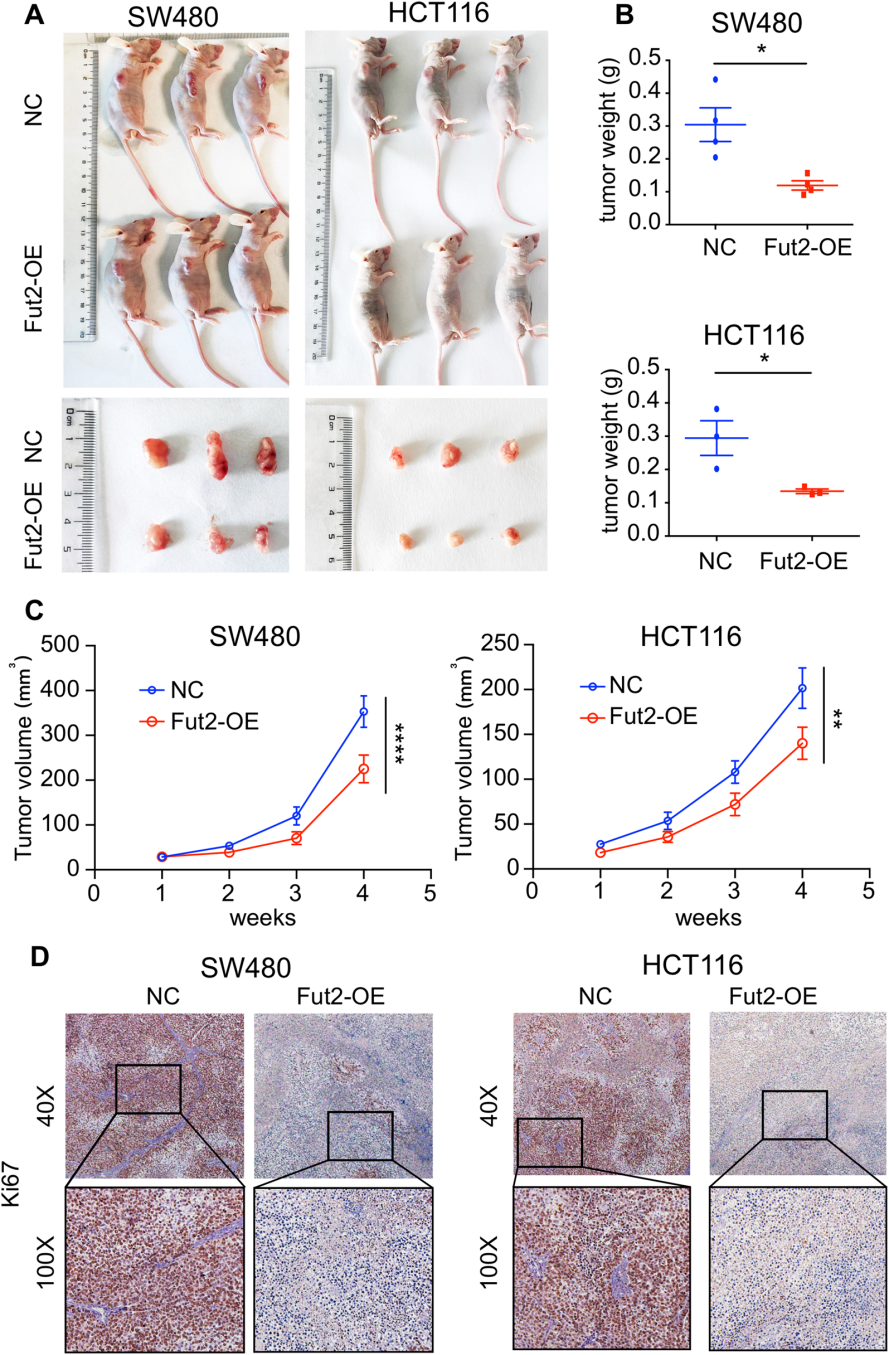
Fut2 inhibits tumor proliferation in xenograft model **A** The typical images of nude mice injected with SW480 and HCT116 cells in control and Fut2-overexpression group. **B** The tumor weight of in control and Fut2-overexpression group of SW480 and HCT116 cells. **C** The tumor volume of in control and Fut2-overexpression group of SW480 and HCT116 cells. **D** The representative image of Ki67 staining of nude mice injected with SW480 and HCT116 cells in control and Fut2-overexpression group. Data are expressed as mean ± SEM. The data come from three independent experiments. In all panels: *p < 0.05, **p < 0.01, ****p < 0.0001. (*SEM* Standard Error of Mean)

In addition, we subcutaneously injected both WT and Fut2-overexpressed SW480 and HCT116 cells into nude mice and measured the tumor weight after 4 weeks. The tumor weights of animals administered Fut2-overexxpressed SW480 and HCT116 cells were significantly lower (p = 0.0132 and p = 0.01, respectively) than those of mice injected with control cells (Fig. 4A, B). The tumor volumes of animals administered Fut2-overexxpressed SW480 and HCT116 cells were significantly lower (p < 0.0001 and p = 0.0071, respectively) than those of mice injected with control cells (Fig. 4C). Immunohistochemical analysis showed that animals injected with Fut2-overexxpressed cells had a lower percentage proliferation of Ki67 + tumor cells than mice administered WT control cells (Fig. 4D).

### Glycoproteomics reveals glycoproteins that may be associated with Fut2-mediated CRC tumorigenesis

**Fig. 5:**
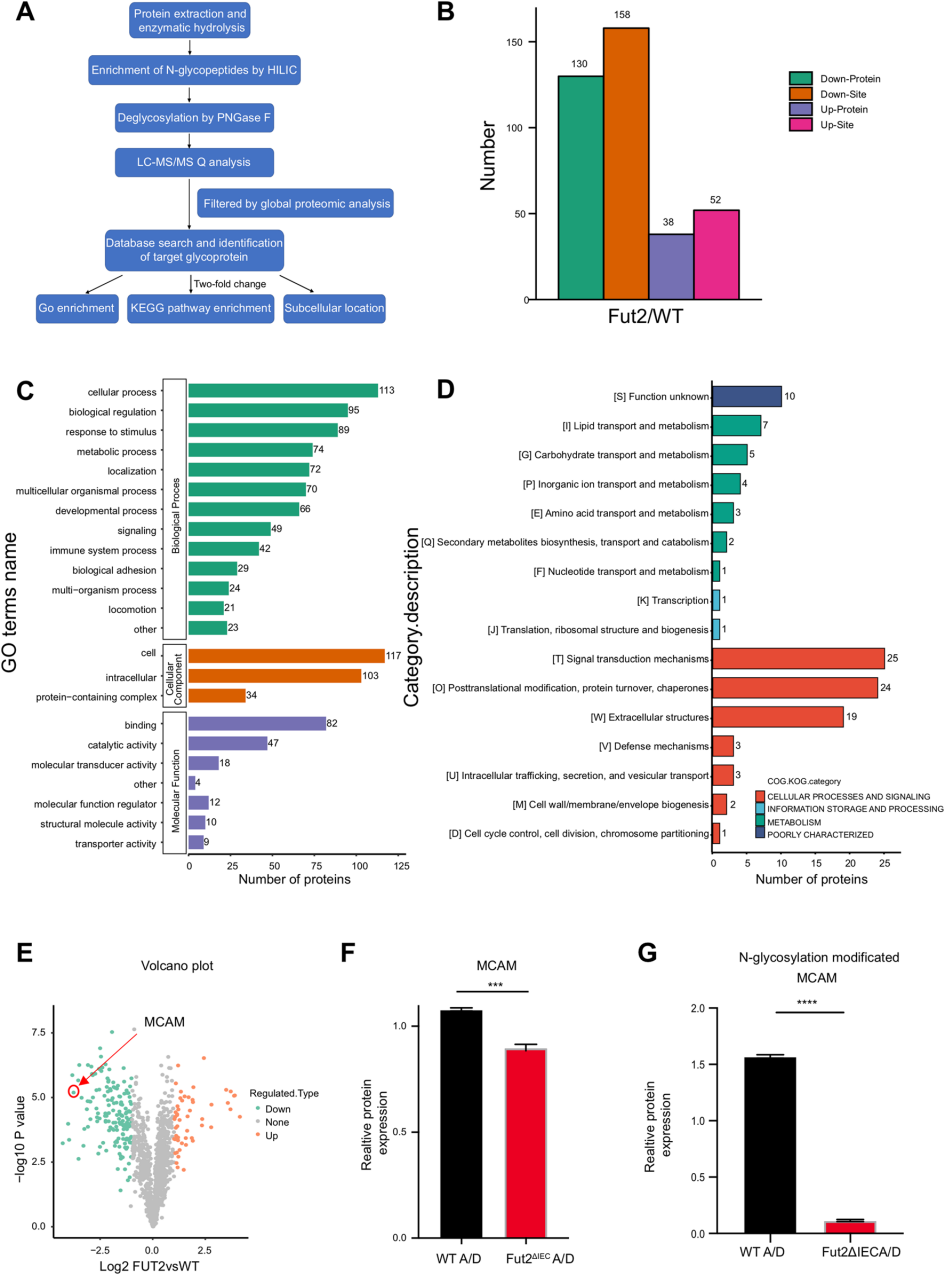
Glycoproteomics reveals glycoproteins that may be associated with Fut2-mediated CRC tumorigenesis **A** Schematic illustration of our systems biology approach to identify N-glycoproteins mediating the effects of Fut2 on CRC metastasis. **B** The number glycoproteins with fold change over 2.0 in WT vs. *Fut2*^△IEC^ mice by proteomic analysis. **C** The differentially expressed glycoproteins by GO analysis in WT vs. *Fut2*^△IEC^ mice. **D** The differentially expressed glycoproteins by COG analysis in WT vs. *Fut2*^△IEC^ mice. **E** The differentially N-glycosylation expressed glycoproteins presented by the volcano plot in WT vs. *Fut2*^△IEC^ mice. **F** The relative protein level of MCAM in colon tissues from WT vs. *Fut2*^△IEC^ mice by proteomic analysis. **G** The relative N-glycosylation expression level of MCAM in colon tissues from WT vs. *Fut2*^△IEC^ mice by glycoproteomic analysis. Data are expressed as mean ± SEM. The data come from three independent experiments. In all panels: ***p < 0.001, ****p < 0.0001. (*CRC* colorectal cancer, *WT* wild type, *MCAM* melanoma cell adhesion molecule, *SEM* Standard Error of Mean)

Proteomics and N-glycoproteomics (LC-MS/MS technology) were carried out to compare the protein and glycoprotein expression levels in the tumors of *Fut2*^△IEC^ mice and the control mice. A schematic illustration of the proposed method is shown (Fig. 5A). Thus, 630 N-glycosylation sites in 428 glycoproteins were identified using quantitative information. Among these glycoproteins, 130 decreased and 38 increased by more than two-fold change (p < 0.05). (Fig. 5B). The differentially expressed glycoproteins identified by Gene Ontology (GO) analysis were implicated in ‘cellular process’, ‘cell component’, ‘binding function’ and so on (p < 0.05). Cellular component analysis by GO revealed that differentially expressed proteins were enriched in ‘cell’ and ‘intracellular’ terms (Fig. 5C). In addition, Clusters of Orthologous Groups analysis showed that differentially expressed glycoproteins were mainly enriched in signal transduction mechanisms and posttranslational modifications (Fig. 5D). To further determine the detailed glycoprotein, the volcano plot showed that MCAM was one of the five glycoproteins whose N-glycosylation expression was the most downregulated (Fig. 5E). MCAM is a cell adhesion molecule associated with tumor progression [20]. Cellular component analysis showed that MCAM belongs to the ‘cell’ term. Proteomics revealed that MCAM in *Fut2*^△IEC^ mice was down-regulated 1.2 times compared to that in control mice (Fig. 5F). However, glycoproteomics revealed that N-glycosylation of MCAM was downregulated 15 times more in *Fut2*^△IEC^ mice than in the control mice, which is much larger than the multiple of its own change (Fig. 5G).

### Fut2 inhibits colon cancer via fucosylation of MCAM

**Fig. 6:**
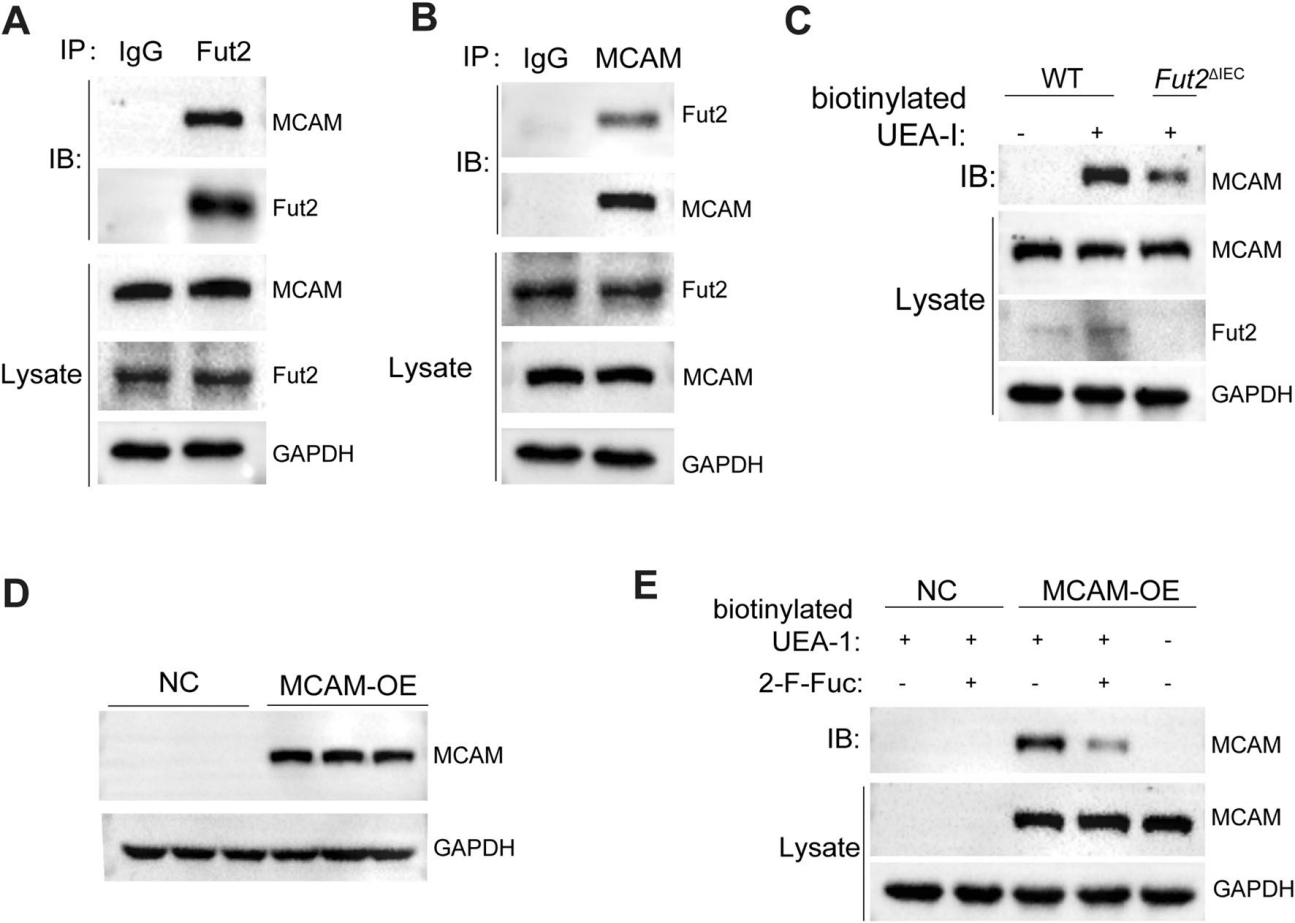
Fut2 interacts with MCAM and mediates the fucosylation of MCAM. **A**, **B** Co-immunoprecipitation experiments showed structural interaction between Fut2 and MCAM. **C** Immunoprecipitation experiments showed the expression of MCAM with or without biotinylated UEA-I in colon tissues of WT and *Fut2*^△IEC^ mice. **D** The typical western blot image of MCAM expression in vector and MCAM overexpression SW480 cell line with Fut2 overexpressed. **E** Immunoprecipitation experiments showed the expression of MCAM with or without biotinylated UEA-I in control and MCAM overexpression SW480 cell line with Fut2 overexpressed (with or without peracetylated 2-F-Fuc treated). Data are expressed as mean ± SEM. The data come from three independent experiments. (*MCAM* melanoma cell adhesion molecule, *UEA-I* Ulex Europaeus Agglutinin-I, *WT* wild type, *2-F-Fuc* 2-fluorofucose, *SEM* Standard Error of Mean)

The detailed mechanism of the differential glycoproteins involved in Fut2-mediated CRC was further studied. Immunoprecipitation experiments in colon tissues from WT mice further demonstrated the interaction between Fut2 and MCAM (Fig. 6A, B). The effect of Fut2 on MCAM was further supported by colon tissues from *Fut2*^△IEC^ and control mice treated with AOM/DSS. In *Fut2*^△IEC^ mice, the glycosylation level of MCAM was lower than that in the control mice (Fig. 6C). Furthermore, MCAM was overexpressed in the Fut2 overexpressed SW480 cell line (Fig. 6D). CoIP studies showed that peracetylated 2-F-Fuc (an inhibitor of fucosyltransferase, 20 μM) could reverse the effect of Fut2 that increases the α-1,2-fucosylation of MCAM (Fig. 6E).

**Fig. 7:**
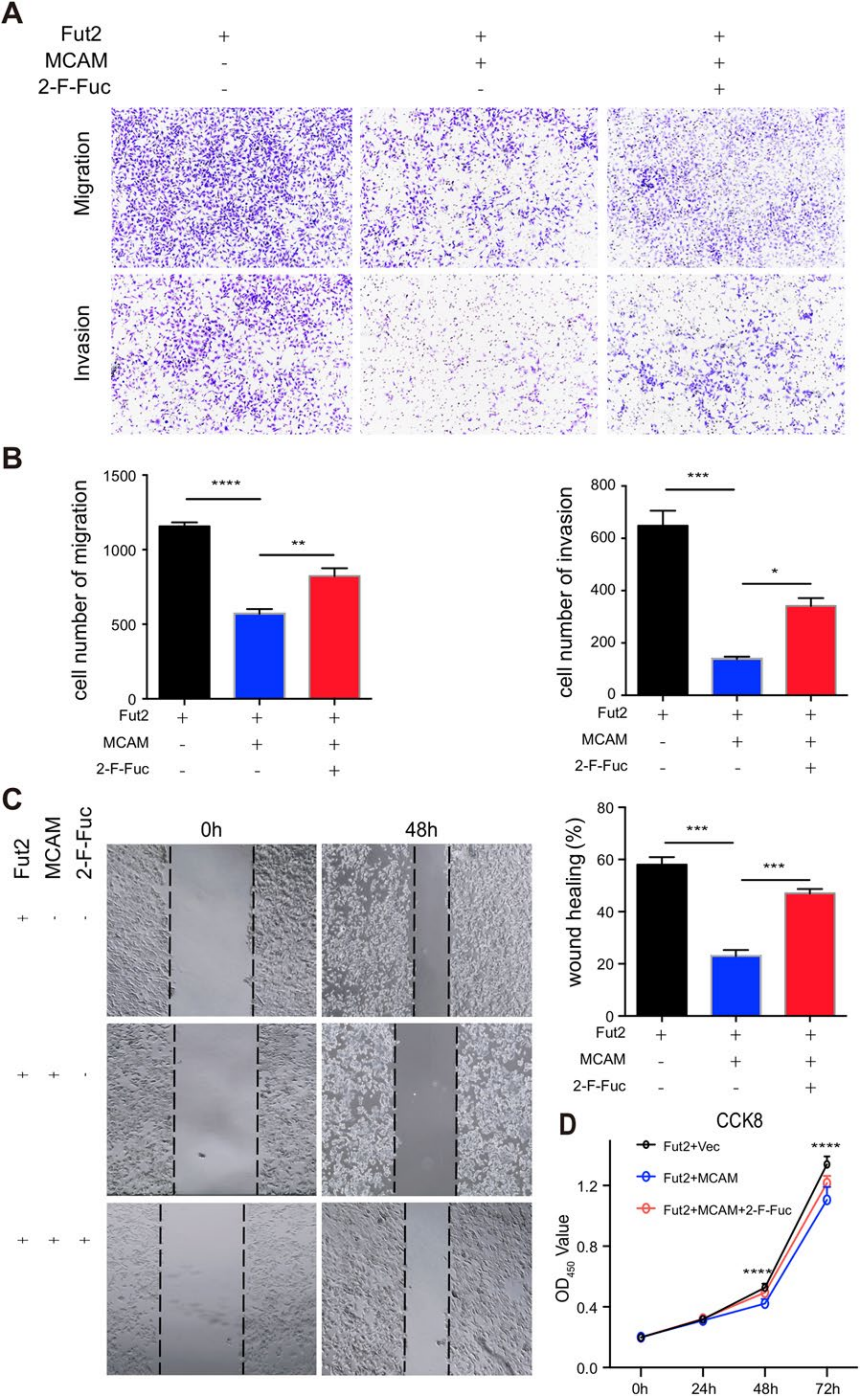
Fut2 inhibits colon cancer via fucosylation of MCAM. **A**, **B** The typical image and corresponding statistical results of transwell migration or invasion assay in control and MCAM overexpression SW480 cell line with Fut2 overexpressed (with or without peracetylated 2-F-Fuc treated). **C** Wound-healing assay at 48 h in control and MCAM overexpression SW480 cell line with Fut2 overexpressed (with or without peracetylated 2-F-Fuc treated). **D** The CCK8 assay at different time points (24 h, 48 h and 72 h) in control and MCAM overexpression SW480 cell line with Fut2 overexpressed (with or without peracetylated 2-F-Fuc treated). Data are expressed as mean ± SEM. The data come from three independent experiments. In all panels: *p < 0.05, **p < 0.01, ***p < 0.001, ****p < 0.0001. (*MCAM* melanoma cell adhesion molecule, *CCK8* Cell Counting Kit-8, *2-F-Fuc* 2-fluorofucose, *SEM* Standard Error of Mean)

Transwell migration, invasion and wound-healing assays revealed that inhibition of MCAM-overexpressing cell migration and invasion was also restored by the inhibitor of fucosyltransferase (Fig. 7A–C). The CCK8 assay revealed that the inhibition of MCAM-overexpressing cell proliferation was restored by an inhibitor of fucosyltransferase (Fig. 7D). These results indicated that down-regulation of MCAM α-1,2-fucosylation reversed the effects of Fut2 overexpression on SW480 cell proliferation, migration, and invasion.

## Discussion

In the present study, decreased expression of Fut2 and α-1,2-fucosylation was observed in patients with CRC. Moreover, we revealed that Fut2 deficiency in the intestinal epithelium exacerbates AOM/DSS-induced CRC, including cell proliferation, migration, and invasive abilities. In addition, prominent down regulation of MCAM fucosylation was detected in colorectal tumor tissues of *Fut2*^△IEC^ mice. Finally, a fucosyltransferase inhibitor repressed the fucosylation modification of MCAM, promoting cell proliferation, invasion and tumor metastasis in Fut2-overexpression cells, indicating that fucosylation of MCAM might be a mediator of Fut2 in CRC. Therefore, our study demonstrated that intestinal epithelium-specific Fut2 deficiency mice were susceptible to AOM/DSS induced CRC by inhibiting the fucosylation of MCAM.

In our study, we demonstrated that the downregulation of Fut2 and α-1,2-fucosylation in the colonic epithelium of CRC patients, and the decreased Fut2 expression level correlated with poor patient survival. The role of Fut2 in other cancers remains controversial. For example, a previous study demonstrated that Fut2 plays important roles in promoting the growth, adhesion, and migration of breast cancer cells [21]. In lung adenocarcinoma (LUAD), it has also been reported that Fut2 promotes cancer metastasis through epithelial-mesenchymal transition initiated by TGF-β/Smad signaling [22, 23]. Additionally, in liver cancer, Fut2 was reported to increase levels of Globo H and enhance cancer cell proliferation [24]. These studies suggest that Fut2 plays an important role in promoting the development of the above-mentioned cancers; however, there are few reports on the role of Fut2 in CRC. Only one study reported that downregulating the transcription of caudal-type homeobox 2 (a tumor suppressor) resulted in a decrease in the transcription level of Fut2, thereby contributing to colon cancer cells metastasis [25]. This study indicated that the decreased levels of Fut2 promoted colon cancer metastasis, which was similar to our results. Therefore, the role of Fut2 in cancer development may be tissue specific.

The abnormal proliferation, migration, and metastasis of tumor cells is a hallmark of tumor pathology during tumor progression [26, 27]. In our study, we demonstrated that Fut2 deficiency enhanced the promotion of AOM/DSS-induced CRC. In addition, overexpression of Fut2 inhibited cell proliferation, invasion and tumor metastasis in colon cancer cells, both in vivo and in vitro. However, other researchers found that silencing Fut2 suppressed cell migration, whereas Fut2 overexpression increased cell migration, invasion and metastasis in breast cancer [21]. Furthermore, Deng et al. found that increased expression of Fut2 has been observed in LUAD, and Fut2 enhanced cell migration and invasion of LUAD cell lines [22]. The different roles of Fut2 in cancers may be due to differences in the tissues and organs. However, the specific mechanism by which Fut2 regulates CRC remains unclear.

The main role of Fut2, a vital α-1,2-fucosyltransferase, is to regulate fucosylation of cells or specific proteins [13]. Our previous study demonstrated that Fut2 mediates fucosylation of intestinal epithelial cells to protect intestinal barrier function and relieve intestinal inflammation [12]. With regard to specific proteins, a study has found that loss of Fut2-mediated mucin O-glycosylation of Muc5ac impairs gastric mucosal binding of *H. pylori* BabA adhesin [16]. In addition, Fut2 can also modulate the glycosylation profile of mucus, thereby regulating the intestinal epithelial barrier functions [17]. Accordingly, we determined whether Fut2 is involved in CRC by regulating fucosylation of certain key proteins.

To determine the most related glycosylated proteins that mediate the effects of Fut2 on CRC development, proteomics and N-glycoproteomics (LC-MS/MS technology) were used to compare the proteins and glycoproteins expression levels in control and *Fut2*^△IEC^ mice. The five most significantly downregulated glycoproteins at the N-glycosylation level were Cathepsin C, Folate hydrolase 1, N-acylsphingosine amidohydrolase 1, Tissue factor and MCAM. MCAM is a cell adhesion molecule that has been associated with CRC progression. However, the other four glycoproteins were not that related with tumorigenesis. MCAM has been considered to be associated with tumor development in human malignant melanoma [20, 28]. A study reported that MCAM is essential for the survival and transformation of hepatocellular carcinoma (HCC) by inducing the translation initiation and transcriptional activities of c-Jun/c-Fos [29]. In contrast, reduced MCAM expression has been revealed to stimulate tumorigenesis and cancer stemness in CRC by activating the Wnt/β-catenin signaling pathway [30]. Another study also demonstrated that MCAM deficiency in human CRC cells significantly promotes tumor progression, further confirming its tumor-suppressive roles in CRC [31]. Therefore, we determined whether MCAM is the key protein that mediates the effects of Fut2 on CRC development.

We first demonstrated the structural interaction between Fut2 and MCAM using CoIP studies, which indicated that there may be a functional interaction between them. After using an inhibitor of fucosyltransferase peracetylated, 2-F-Fuc, the glycosylation modification effect of Fut2 on MCAM was significantly weakened, reflecting the regulatory effect of Fut2 on MCAM glycosylation. We further verified the influence of MCAM on the involvement of Fut2 in inhibiting CRC. In the Fut2-overexpressed SW480 cell line, the results showed that MCAM overexpression extended the inhibitory effects on migration, invasion, and cell proliferation. More importantly, using an inhibitor of fucosyltransferase could restored the inhibitory effect of SW480 caused by the overexpression of MCAM. Taken together, these results demonstrated that Fut2 inhibits the progression of CRC by promoting the glycosylation of MCAM.

In summary, our findings demonstrate that a deficiency of fucosylation due to a Fut2 mutation leads to low fucosylation of MCAM, followed by CRC progression and metastasis. We believe that Fut2 and MCAM are promising compounds in CRC therapy.

## Conclusions

Our results revealed that intestinal epithelium-specific Fut2 deficiency mice were susceptible to AOM/DSS-induced CRC by inhibiting the fucosylation of MCAM. The regulation of fucosylation may be a potential therapy for CRC, especially in patients with Fut2 gene defects.

## Supplementary Information

**Additional file 1**: **Table S1.** Sequences of Primers Used for Real-Time Quantitative PCR

**Additional file 2**: Supplementary methods.

## Data Availability

The datasets used and/or analyzed during the current study are available from the corresponding author on reasonable request.

## Abbreviations

AOM: Azoxymethane
AUC: Area under the curve
CDX2: Caudal type homeobox 2
COG: Clusters of orthologous groups
CoIP: Co-immunoprecipitation
CRC: Colorectal cancer
DSS: Dextran sulfate sodium
EMT: Epithelial-mesenchymal transition
Fut2: Fucosyltransferase 2
GO: Gene ontology
HCC: Hepatocellular carcinoma
LC-MS/MS: Liquid chromatography tandem mass spectrometry
LUAD: Lung adenocarcinoma
MCAM: Melanoma cell adhesion molecule
PCR: Polymerase chain reaction
SPF: Specific pathogen free
TCGA: The cancer genome atlas
UEA-I: Ulex Europaeus agglutinin I
WT: Wild type
