# Supplementary material for: Intestinal epithelium-specific Fut2 deficiency promotes colorectal cancer through down-regulating fucosylation of MCAM
Source: J Transl Med. 2023 Feb 4;21:82. doi: 10.1186/s12967-023-03906-0 (PMC9899399; doi:10.1186/s12967-023-03906-0)
Supplement: Supplementary file 1 — Additional file 1: Table S1. Sequences of Primers Used for Real-Time Quantitative PCR [file 12967_2023_3906_MOESM1_ESM.docx]

**Table S1**

**Sequences of Primers Used for Real-Time Quantitative PCR**

| **Genes** | **Primer F** | **Primer R** |
| --- | --- | --- |
| **mFUT2** | **ACCACAGCCAGAAGAGGATTG** | **GAAAGGTACCTGGGCACTCG** |
| **hFut2** | **CTACCACCTGAACGACTGGATG** | **AGGGTGAACTCCTGGAGGATCT** |
| **mGAPDH** | **CATGGCCTTCCGTGTTCCTA** | **TACTTGGCAGGTTTCTCCAGG** |
| **hGAPDH** | **CCCCACTTGATTTTGGAGGGA** | **AGGGCTGCTTTTAACTCTGGT** |
|  |  |  |
