# Supplementary material for: Intestinal epithelium-specific Fut2 deficiency promotes colorectal cancer through down-regulating fucosylation of MCAM
Source: J Transl Med. 2023 Feb 4;21:82. doi: 10.1186/s12967-023-03906-0 (PMC9899399; doi:10.1186/s12967-023-03906-0)
Supplement: Supplementary file 2 — Additional file 2: Supplementary methods. [file 12967_2023_3906_MOESM2_ESM.docx]

**Proteomic analysis**

*Protein Extraction*

The sample was grinded with liquid nitrogen into cell powder and then transferred to a 5-mL centrifuge tube. After that, four volumes of lysis buffer (8 M urea, 1% protease inhibitor cocktail) was added to the cell powder, followed by sonication three times on ice using a high intensity ultrasonic processor (Scientz). (Note: For PTM experiments, inhibitors were also added to the lysis buffer, e.g. 3 μM TSA and 50 mM NAM for acetylation, 1% phosphatase inhibitor for phosphorylation). The remaining debris was removed by centrifugation at 12,000 g at 4 °C for 10 min. Finally, the supernatant was collected and the protein concentration was determined with BCA kit according to the manufacturer’s instructions.

*Trypsin Digestion*

For digestion, the protein solution was reduced with 5 mM dithiothreitol for 30 min at 56 °C and alkylated with 11 mM iodoacetamide for 15 min at room temperature in darkness. The protein sample was then diluted by adding 100 mM TEAB to urea concentration less than 2 M. Finally, trypsin was added at 1:50 trypsin-to-protein mass ratio for the first digestion overnight and 1:100 trypsin-to-protein mass ratio for a second 4 h-digestion. Finally, the peptides were desalted by C18 SPE column.

*TMT Labeling*

Tryptic peptides were firstly dissolved in 0.5 M TEAB. Each channel of peptide was labeled with their respective TMT reagent (based on manufacturer’s protocol, ThermoFisher Scientific), and incubated for 2 hours at room temperature. Five microliters of each sample were pooled, desalted and analyzed by MS to check labeling efficiency. After labeling efficiency check, samples were quenched by adding 5% hydroxylamine. The pooled samples were then desalted with Strata X C18 SPE column (Phenomenex) and dried by vacuum centrifugation.

*HPLC Fractionation*

The sample was fractionated into fractions by high pH reverse-phase HPLC using Agilent 300 Extend C18 column (5 μm particles, 4.6 mm ID, 250 mm length). Briefly, peptides were separated with a gradient of 2% to 60% acetonitrile in 10 mM ammonium bicarbonate pH 10 over 80 min into 80 fractions. Then, the peptides were combined into 9 fractions and dried by vacuum centrifuging.

*Affinity Enrichment*

Bio-material-based PTM enrichment (for N-glycosylation): Tryptic peptides were redissolved in 200 μL washing buffer (80% ACN, 5% TFA) and then loaded onto the column and then washed with washing buffer for three times. Glycopeptides were eluted with 0.1% TFA, 50mM ammonium bicarbonate and 50% ACN for two times. The eluted glycopeptides were dried in the Speedvac and redissolved with 50 μL of 50 mM ammonium bicarbonate solution dissolved in H2O18. After adding 2 μL of PNGase F glycosidase, the digestion was performed at 37°C overnight. Finally, deglycopeptides were desalted using C18 Zip Tips according to the manufacturer’s instructions and then dried for further MS analysis.

*LC-MS/MS Analysis*

The tryptic peptides were dissolved in solvent A (0.1% formic acid, 2% acetonitrile/ in water), directly loaded onto a home- made reversed-phase analytical column (25-cm length, 75 μm i.d.). Peptides were separated with a gradient from 5% to 25% solvent B (0.1% formic acid in 90% acetonitrile) over 60 min, 25% to 35% in 22 min and climbing to 80% in 4 min then holding at 80% for the last 4 min, all at a constant flowrate of 450 nL/min on an EASY-nLC 1200 UPLC system (ThermoFisher Scientific). The separated peptides were analyzed in Q ExactiveTM HF-X (ThermoFisher Scientific) with a nano-electrospray ion source. The electrospray voltage applied was 2.0 kV. The full MS scan resolution was set to 60,000 for a scan range of 350–1600 m/z. Up to 20 most abundant precursors were then selected for further MS/MS analyses with 30 s dynamic exclusion. The HCD fragmentation was performed at a normalized collision energy (NCE) of 28%. The fragments were detected in the Orbitrap at a resolution of 30,000. Fixed first mass was set as 100 m/z. Automatic gain control (AGC) target was set at 1E5, with an intensity threshold of 3.3E4 and a maximum injection time of 60 ms.

The resulting MS/MS data were processed using MaxQuant search engine (v.1.6.15.0). Tandem mass spectra were searched against the human SwissProt database (20422 entries) concatenated with reverse decoy database. Trypsin/P was specified as cleavage enzyme allowing up to 2 missing cleavages. The mass tolerance for precursor ions was set as 20 ppm in first search and 5 ppm in main search, and the mass tolerance for fragment ions was set as 0.02 Da. Carbamidomethyl on Cys was specified as fixed modification, and acetylation on protein N-terminal and oxidation on Met were specified as variable modifications. FDR was adjusted to < 1%.
